# Fahrenheit and Centigrade

**Published:** 1899-02

**Authors:** 


### Fahrenheit and Centigrade.

In a recent lecture Prof. Billings emphasized the importance of being familiar with the several temperature scales. The following from the *Dietetic and Hygienic Gazette* may aid in making the conversion;

From Centigrade to Fahrenheit,
'Tis easy to divine—
You first must use arithmetic
And multiply by nine.

The answer now divide by five,
And then you have in view
The very number that you seek,
By adding thirty-two.

From Fahrenheit to Centigrade,
However, it is plain—
You first must take the thirty-two
And multiply again;
But this time only by the five,
And then you draw a line
Straight up and down, in order that
You may divide by nine. —*The Corpuscle.*

